# Recovery Phase in the Chain of Survival in Cardiac Arrest Patients: Scoping Review

**DOI:** 10.21315/mjms-10-2025-730

**Published:** 2026-02-28

**Authors:** Kameliana, Titin Andri Wihastuti, Suryanto

**Affiliations:** 1 Master of Nursing Study Program, Faculty of Health Sciences, Universitas Brawijaya, Malang, East Java, Indonesia; 2 Department of Nursing, Faculty of Health Sciences, Universitas Brawijaya, Malang, East Java, Indonesia

**Keywords:** cardiac arrest, Chain of Survival, recovery, outcome, survival, rehabilitation

## Abstract

Sudden cardiac arrest (SCA) remains a major global health concern, with an estimated 300,000 to 400,000 deaths annually in the United States and around two million worldwide. In 2020, the American Heart Association (AHA) updated the Chain of Survival by adding a new component recovery to highlight the importance of post-resuscitation care. However, the implementation of this recovery phase remains limited and requires further exploration. This study aimed to review the implementation of the recovery phase within the Chain of Survival. A scoping review design was employed, with literature searches conducted in ScienceDirect, ProQuest, Sage, and PubMed databases using the keywords (“Cardiac Arrest”) AND (“Chain of Survival”) AND (“Recovery”) AND (“Rehabilitation”). The inclusion criteria included articles published within the last five years, written in English, and available in full-text open access format. Of the 330 articles initially identified, 13 met the eligibility criteria and were analysed using the Joanna Briggs Institute framework, with the findings reported in accordance with the PRISMA-ScR guidelines. Three major themes emerged: (i) multidimensional challenges among survivors, including physical, cognitive, psychological, and social impairments; (ii) multidisciplinary interventions, such as the ROCK and SCARF programmes, which were shown to improve quality of life, reduce fatigue, and enhance independence; and (iii) factors influencing recovery, including biomarkers, prognostic scores, and socioeconomic status. The recovery phase is a crucial yet often neglected component of the Chain of Survival. Post-cardiac arrest care should adopt a comprehensive and integrated approach involving evidence-based rehabilitation, psychological support, and family counselling to achieve optimal recovery and long-term quality of life.

## Introduction

Cardiac arrest is a significant global health problem. Sudden cardiac arrest (SCA) is one of the leading causes of death in the United States, with approximately 300,000 to 400,000 deaths each year, accounting for about 10% of all deaths from heart disease (1). Other studies suggest that the global incidence of cardiac arrest remains high due to the large at risk population, with at least two million cases occurring worldwide annually, making SCA a major global health concern (2).

Research reported in the AHA’s Heart Disease and Stroke Statistics 2020 Update indicates that emergency medical services in the United States respond to more than 347,000 adults and over 7,000 children (under 18 years of age) with out-of-hospital cardiac arrest (OHCA) each year. Meanwhile, in-hospital cardiac arrest (IHCA) is estimated to occur in 9.7 per 1,000 adult inpatients (approximately 292,000 cases annually) and 2.7 paediatric cases per 1,000 paediatric inpatients (3). Based on research conducted in Qatar, 72.8% occurred outside hospitals (Out-Hospital Cardiac Arrest/OHCA) and 27.2% occurred inside hospitals (4). In addition, research conducted in Germany reports that the survival rate for OHCA ranges from 0% to 18%. In contrast, the survival rate for IHCA ranges from 15% to 34%. Overall, the average survival rate is approximately 8% (5).

**Figure 1:**
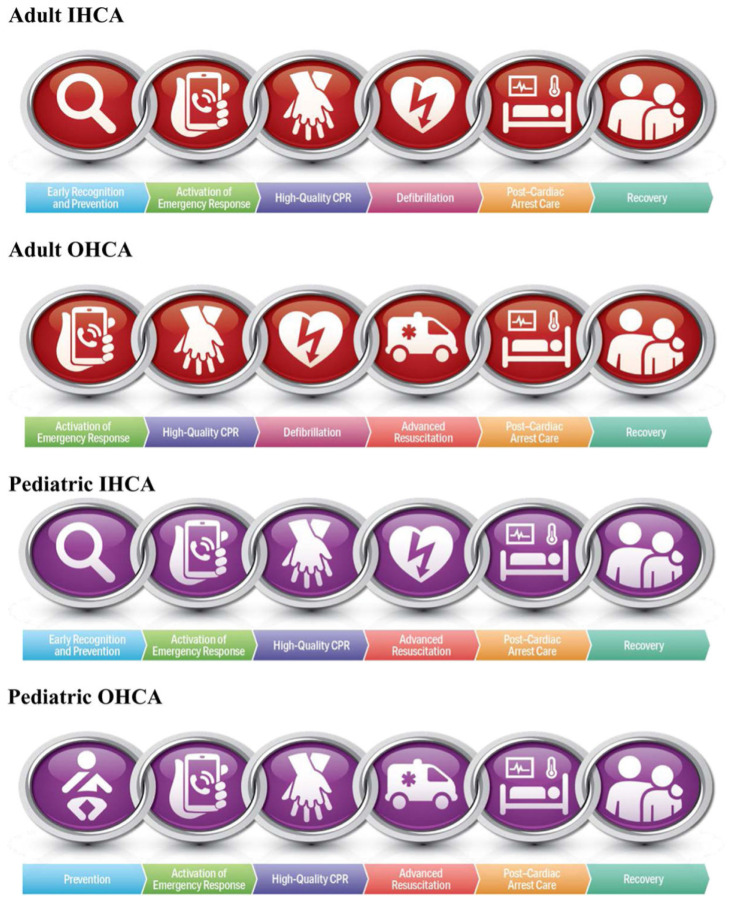
AHA 2020 Chain of Survival

As part of its efforts to improve survival rates among cardiac arrest patients, the AHA developed the Chain of Survival concept. This framework is regularly updated based on the latest evidence. In the most recent update in 2020, the Chain of Survival was expanded to include six components for both IHCA and OHCA, applicable to both adults and children. One of the significant changes in the 2020 Chain of Survival is the addition of the recovery component (3). The Chain of Survival for IHCA and OHCA patients based on the 2020 AHA guidelines is shown in Figure 1.

To date, publications describing the implementation of the recovery component in the Chain of Survival for cardiac arrest patients remain limited. This is reflected in research findings indicating that the recovery component accounts for less than 8% of the overall reported outcomes. This imbalance reflects a greater focus on the early phases, such as Early Recognition and Prevention and Post-cardiac Arrest Care, while the long-term recovery aspect receives less attention, even though it plays an important role in the quality of life of patients after cardiac arrest (6).

The recovery phase represents both a pathway to healing and a structured plan for survival, encompassing treatment, monitoring, and rehabilitation. It aims to address the physical and psychological consequences of cardiac arrest, including anxiety, depression, post-traumatic stress, and fatigue experienced by survivors and their caregivers. It also includes multimodal rehabilitation care for physical, neurological, cardiopulmonary, and cognitive impairments before discharge from the hospital. In addition, cardiac arrest survivors also receive comprehensive and multidisciplinary discharge planning, including medical and rehabilitation treatment recommendations and expectations for returning to activities or work (3).

Other studies also indicate that postcardiac arrest recovery represents the final stage of the Chain of Survival, focusing on reintegrating patients into society with the support of healthcare providers and caregivers. This process can be challenging, as recovery often takes considerable time at least 11 weeks for noticeable improvement in self care abilities, while most cognitive improvements tend to occur within the first three months. Additional burdens arise when caregivers face financial, social, emotional, or health problems. Therefore, early and coordinated planning, as well as a continuous evaluation system, are needed to optimise patient recovery and utilise knowledge from clinical experience to improve services (7).

However, to date, no published research has specifically explored the implementation of the recovery component in the Chain of Survival for cardiac arrest patients. By gaining a deeper understanding of this issue, this study aims to provide guidance for implementing the recovery phase in cardiac arrest care, with the ultimate goal of improving survival rates and long-term outcomes for patients who have experienced cardiac arrest. Therefore, the purpose of this scoping review is to map the current implementation of the recovery phase in cardiac arrest patients.

## Methods

### Study Design

**Figure 2:**
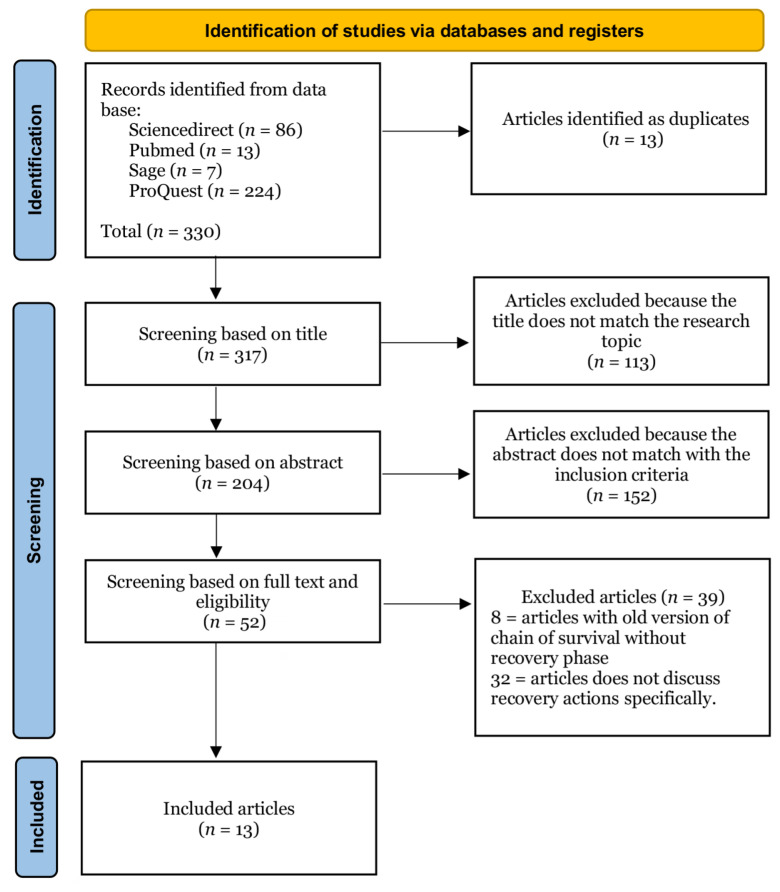
PRISMA-ScR Flowchart

This study was conducted as a scoping review to explore the implementation of the recovery phase within the Chain of Survival for cardiac arrest patients. Although the review protocol was not formally registered, the study followed the Joanna Briggs Institute (JBI) methodological framework and was reported in accordance with the PRISMA-ScR checklist (Figure 2). Data charting and synthesis were carried out independently by three reviewers, and any discrepancies were resolved through discussion until consensus was reached.

### Eligibility Criteria

The inclusion and exclusion criteria were determined using the PCC (Population, Concept, Context) framework to define the scope of the review and the research questions, as shown in Table 1.

Inclusion criteria were: i) published within the last five years (2020–2025); ii) original research (not review or commentary); iii) written in English, full-text, and open access; iv) relevant to the topic of cardiac arrest recovery within the Chain of Survival.

Exclusion criteria were: i) non-English publications or books; ii) lacking abstracts or full-text access; iii) duplicates; iv) focused on topics outside the recovery phase of the Chain of Survival.

### Information Sources and Search Strategy

A comprehensive literature search was carried out in four electronic databases: ScienceDirect, ProQuest, SAGE, and PubMed. The search was conducted between 31 July and 3 August 2025. The Boolean operator “AND” was applied to combine the following keywords: (“Cardiac Arrest”) AND (“Chain of Survival”) AND (“Recovery”) AND (“Rehabilitation”). The search strategy was tailored to match the specific syntax and indexing system of each database. All identified records were exported to reference management software for organisation and documentation. Duplicate entries were removed before proceeding to the screening stage.

### Study Selection Process

The study selection process was conducted in two sequential stages. First, all identified titles and abstracts were screened according to the predefined inclusion criteria. Subsequently, the full texts of studies considered potentially eligible were independently reviewed by three researchers. Any disagreements were discussed collectively and resolved through consensus. A total of 330 articles were initially retrieved from four electronic databases: 224 from ProQuest, seven from SAGE, 13 from PubMed, and 86 from ScienceDirect. After removing 13 duplicates, 317 articles remained for screening. Following title, abstract, and full-text assessment, 13 articles met all eligibility criteria and were included in this review. The detailed selection process is presented in Figure 1.

### Data Extraction

A standardised data charting form was developed to extract relevant information from each included study systematically. Extracted data included: i) author and year of publication; ii) country of origin; iii) study design; iv) sample characteristics; v) outcomes related to recovery implementation; vi) key findings related to rehabilitation, quality of life, and return to function. Data extraction was conducted independently by the three reviewers and verified through cross-checking to ensure consistency and accuracy.[Table t2-03mjms3301_ra]

### Quality Appraisal

Although critical appraisal is not a mandatory component of a scoping review, an assessment of methodological quality was undertaken using the appropriate JBI Critical Appraisal Checklist, selected according to each study design. Studies that fulfilled ≥70% of the JBI criteria were classified as having a low risk of bias, those meeting 50% to 69% were categorised as moderate risk, and those scoring < 50% were considered high risk. Articles employing designs for which no suitable JBI appraisal tool was available (e.g., survey-based descriptive studies) were labelled as “Not Appraised (No suitable JBI tool).” Nevertheless, these studies were retained to ensure a comprehensive mapping of the available evidence.

### Data Synthesis

A narrative thematic analysis was employed to synthesise the findings across the included studies. The extracted data were systematically organised around key themes related to the recovery phase, encompassing the physical, cognitive, psychological, and social domains. The analysis involved iterative steps of familiarisation, coding, and categorisation of key findings into overarching themes. These themes were then discussed among the reviewers and refined to ensure accuracy and conceptual clarity. The final thematic structure is presented in the results section.

## Results

### Study Selection and Characteristics

A total of 13 articles were included in this scoping review after the application of the predefined inclusion and exclusion criteria, in accordance with the PRISMA-ScR guideline. The selected studies were published between 2021 and 2024 and reflected a broad international scope, representing research conducted in Denmark, Canada, Switzerland, South Korea, China, Sweden, Iran, Australia, New Zealand, and England. Most of the included studies adopted quantitative designs such as randomised controlled trials, cohort, and cross-sectional studies, while several employed qualitative or mixed-methods approaches. Overall, the studies explored post-cardiac arrest recovery from multidimensional perspectives encompassing physical, cognitive, emotional and social measures and studying different interventions and predictors regarding long-term effects.[Table t3-03mjms3301_ra]

### Key Findings: Recovery Themes and Influencing Factors

Thematic synthesis of the 13 included studies identified three overarching themes that capture the complexity of the recovery phase after cardiac arrest.

#### Multidimensional Challenges in Post-Cardiac Arrest Recovery

Most of the included studies consistently reported that survivors of cardiac arrest continue to experience persistent, multidimensional challenges long after hospital discharge. These challenges span various domains, including cognitive impairments, prolonged fatigue, anxiety, depression, loss of identity, and increased social dependency. Several studies found that nearly 50% of working-age survivors failed to return to employment due to neurocognitive and emotional deficits (15, 19). Other research highlighted identity disruption and emotional strain among survivors, while their families experienced psychological distress and caregiving burden (8).

Additional findings highlighted the presence of Post-Intensive Care Syndrome (PICS), characterised by symptoms such as depression, anxiety, and sleep disturbances, which in some cases persisted for up to 12 months following the cardiac arrest event (13, 17). Several studies reported insufficient psychological and social follow-up for survivors and their families (10). In contrast, other findings indicated that nearly half of survivors experienced subtle cognitive impairments, particularly affecting executive functioning and visuospatial abilities (20). Collectively, these findings underscore that recovery after cardiac arrest is a complex, multifaceted process involving physical healing, brain function, emotional stability, and social reintegration.

#### Effective Multidisciplinary and Structured Rehabilitation Interventions

Six studies highlighted the critical role of comprehensive, multidisciplinary rehabilitation in improving outcomes following cardiac arrest. One study introduced the ROCK (Return to Work after Cardiac Arrest) programme, which integrates physical, psychological, and social support components to facilitate survivors’ successful reintegration into the workforce (15). Another study reported the success of the SCARF (Survivors of Cardiac Arrest Focused on Fatigue) programme, which reduced fatigue and anxiety through combined physical therapy, counselling, education, and energy management (12).

Additional findings underscored the value of peer support in enhancing survivors’ self understanding and facilitating emotional adaptation (11). Significant functional gains were also observed during inpatient rehabilitation. However, many patients continued to experience dependency in activities of daily living, highlighting the ongoing need for structured long-term follow-up after discharge (9). Other research called for comprehensive, multidisciplinary follow-up that includes psychological and cognitive assessment (10), while structured, evidence-based post-discharge programmes were supported to optimise long-term outcomes. Overall, the evidence demonstrates that integrated, structured rehabilitation and peer-supported care models effectively improve survivors’ quality of life, autonomy, and psychosocial well-being.

#### Biological, Clinical, and Socioeconomic Predictors of Recovery Outcomes

Seven studies identified biological, clinical, and socioeconomic factors that influence recovery trajectories following cardiac arrest. One study reported that patients with lower socioeconomic status experienced significantly poorer outcomes, including lower rates of return of spontaneous circulation (ROSC), reduced survival, and less favourable neurological recovery (16). Another study reported that an elevated neutrophil to monocyte ratio (NMLR) was significantly associated with increased death and worse neurological outcomes, emphasising the fact that the role of systemic inflammation as an important prognostic indicator following cardiac arrest (14). The CASPRI score was also validated as an accurate predictor of neurological outcomes following IHCA, aiding in rehabilitation planning and prognosis communication (18).

Additional findings indicated that patients presenting with initial non-shockable rhythms, such as asystole or pulseless electrical activity (PEA), were more likely to develop symptoms of anxiety and depression (17). Conversely, younger age and shorter lengths of hospital stay were identified as predictors of more favourable RTW outcomes (19). Lower baseline FIM scores were associated with greater functional gains during rehabilitation (9), andhigher education served as a protective factor against cognitive decline (20). Together, these studies highlight that successful recovery is shaped by the interaction between biological resilience and social context, emphasising the need for equitable access to rehabilitation services.

## Discussion

### Problems Arising in the Recovery Phase

The recovery phase following cardiac arrest presents complex, multidimensional challenges that encompass physical, psychological, cognitive, and social domains. In addition, systemic limitations within healthcare services often hinder the provision of structured, long-term support for survivors. For example, Christensen et al. (15) highlight the substantial unmet rehabilitation needs among OHCA survivors. Many individuals experience persistent neurocognitive impairments, emotional disturbances including depression, anxiety, and post-traumatic stress symptoms and difficulties returning to work. Despite these ongoing challenges, post-hospitalisation rehabilitation services are frequently fragmented or insufficient to support long-term recovery adequately. They note that approximately 50% of survivors fail to return to work or must accept job modifications, indicating a gap in providing comprehensive cognitive assessments and individualised rehabilitation plans (15). These findings highlight the challenges of identifying rehabilitation needs and accessing structured services as key obstacles in the recovery phase.

Mion et al. (10) identified a notable gap in the follow-up care of OHCA survivors. Based on survey data collected from patients and their families, the majority of respondents reported that they did not receive comprehensive support after hospital discharge, indicating shortcomings in post-acute care services. Follow-up care was generally limited to appointments with a cardiologist, despite many survivors continuing to experience persistent fatigue, cognitive impairments, and psychological difficulties. In addition, family members reported experiencing emotional trauma and feelings of guilt, often without limited access to sufficient counselling or organised psychosocial support services. This study emphasises that a follow-up system that focuses solely on cardiovascular aspects fails to address the need for comprehensive recovery (10). This highlights the lack of a comprehensive follow-up service structure and the need for multidisciplinary integration in the recovery phase.

Dainty et al. (8) highlight the complexity of psychological challenges faced by survivors through a qualitative approach. Their findings reveal that cardiac arrest survivors frequently describe profound feelings of identity loss, persistent anxiety, and an ongoing fear of experiencing another cardiac event. Survivors also reported feelings of alienation, largely because the current healthcare system provides limited space for them to express and process their emotional experiences openly. Furthermore, spouses and other family members frequently emphasised persistent fears of recurrence, along with the substantial physical and emotional burden associated with caregiving responsibilities. This study concludes that the concept of “recovery” used in the healthcare system is still limited to physical survival, whereas patients and families need recovery of meaning in life, a sense of security, and social connection.

Vincent et al. (13) further reinforce this perspective by documenting a high prevalence of PICS among cardiac arrest survivors. Their findings indicate that survivors commonly experience persistent physical symptoms, such as fatigue and generalised weakness, alongside psychological disturbances including depression, anxiety, and sleep disorders that may continue for up to 12 months following hospital discharge. PICS has been shown to be associated with a decline in quality of life and an inability to return to work. This problem shows that recovery after cardiac arrest is not just an acute phase, but a long-term process that requires systematic intervention.

Joshi et al. (12) also identified fatigue as the most dominant and disruptive symptom in life after cardiac arrest. Many survivors described experiencing profound exhaustion that was disproportionate to their level of physical activity, significantly limiting their ability to engage in social interactions and return to occupational roles. Research findings indicate that interventions for cardiac arrest survivors should not only focus on physical recovery but must also address psychological, social, and occupational aspects, as the problems experienced are complex and interrelated. Meanwhile, Nanjayya et al. (9) focused on inpatient rehabilitation outcomes and found that although patients showed significant improvements in motor function, cognitive problems and dependence on caregivers remained high. This indicates that physical recovery is achieved more quickly than cognitive recovery, leaving many patients unprepared for independent activities after discharge.

Arestedt et al. (17) highlight differences in quality of life outcomes based on the initial cardiac rhythm. Their findings indicate that patients presenting with non-shockable rhythms, such as PEA or asystole, tend to experience more severe psychological disturbances and report lower overall quality of life compared to those with shockable rhythms. This condition shows that although initial clinical factors are important, the accompanying psychosocial impact is also significant and needs attention in the recovery phase. Additionally, Zarifkar et al. (20) also found that cognitive dysfunction and changes in brain network connectivity are significant issues that hinder recovery. Using functional MRI, they identified dysfunction in the frontoparietal and visual cortical regions, which was associated with impairments in executive functioning, memory, and visuospatial processing. These findings offer a neurobiological explanation for the psychological symptoms such as depression and anxiety frequently reported by cardiac arrest survivors.

#### Intervention in the Recovery Phase

A number of studies have emphasised the importance of thorough interventions which include physical, psychological, social and occupational aspects to promote optimal recovery after cardiac arrest. In this context, Christensen et al. (15) introduced the ROCK protocol as a structured multidisciplinary intervention model designed to facilitate survivors’ reintegration into daily life and employment. This programme combines the roles of psychologists, physical therapists, occupational physicians, and social workers to help survivors gradually return to work. This intervention focuses on physical strengthening, cognitive rehabilitation, and psychosocial support. This study shows that this model not only increases the rate of return to work (RTW) but also improves patients’ quality of life and self-confidence.

Wagner et al. (11) contribute an important perspective on the role of peer support in post-cardiac arrest rehabilitation. Their findings provide strong evidence that incorporating peer support into rehabilitation programmes yields meaningful psychological benefits. Participants reported that involvement in group based rehabilitation allowed them to feel understood, validated, and less isolated, while also creating a safe space to share recovery experiences with fellow survivors. Through group conversations, learning about hypoxic brain injury and coping mechanisms for anxiety. This increased their confidence, reduced anxiety, and strengthened their commitment to the recovery process. This community-based intervention is evidence that social support can accelerate psychological recovery (11). This perspective is reinforced by Dainty et al. (8), who advocate for recovery approaches that extend beyond medical stabilisation and focus on restoring identity and meaning in life. They recommend psychosocial interventions grounded in peer support and reflective group processes, enabling survivors to make sense of the changes they experience following cardiac arrest and to reconstruct their sense of self.

Joshi et al. (12) conducted the SCARF programme, which is a five-day residential rehabilitation programme followed by remote follow-up. The programme includes education on fatigue, energy management strategies, light physical exercise, and cognitive behavioural therapy. The findings showed a remarkable fatigue decline, together with overall quality of life improvement and emotional functioning increase. The work highlights the availability of services which build a protective emotional space and provide structured education to help patients rebuild their sense of security and meaning in life. Notably, the intervention was shown to be both feasible and positively received by participants, highlighting its potential value in post-cardiac arrest care.

Vincent et al. (13) concluded that PICS is a common condition among cardiac arrest survivors and stressed the importance of routine screening and early intervention. They argue that proactive identification and timely management are essential to prevent or mitigate the longterm physical and psychological consequences associated with PICS. They also proposed the need for long-term multidisciplinary follow-up, including physical and cognitive rehabilitation and psychosocial support for cardiac arrest survivors to help patients adapt to life after cardiac arrest. Mion et al. (10) also proposed structured multidimensional follow-up after patients are discharged from the hospital. They emphasised the need to establish a structured post-discharge pathway that extends beyond routine medical follow-up to include psychological assessment, family counselling, and comprehensive physical and cognitive rehabilitation. Survivors in this study expressed a clear desire for a sustainable recovery programme, one that addresses not only their physical condition but also supports their mental well-being and social reintegration.

Arestedt et al. (17) suggest an intervention approach that is sensitive to early rhythms. Non-shockable patients require more intensive psychological monitoring and rehabilitation plans that emphasise mental support and social reintegration. They recommend needs-based rehabilitation programmes that combine physical and psychological interventions to improve survivors’ quality of life. Additionally, Zarifkar et al. (20) propose that functional MRI (fMRI) may serve as a valuable neurocognitive screening tool. They recommend incorporating neuroimaging assessments into rehabilitation protocols to help predict cognitive outcomes and monitor recovery trajectories more accurately over time. Thus, interventions can be personalised according to each patient’s brain network profile, allowing cognitive rehabilitation strategies to be more accurately targeted. They emphasise the importance of an individualised approach grounded in the patient’s specific brain connectivity patterns, enabling more precise and potentially effective recovery planning.

This is in line with research by Joshi et al. (21) which found that various rehabilitation interventions, both hospital-based (inpatient rehabilitation) and community-based, consistently provide benefits for the physical and neurological recovery of cardiac arrest survivors. This perspective strengthens the view that recovery should be recognised as a core component of the Chain of Survival, rather than merely a supplementary phase following acute treatment. Structured rehabilitation enables patients to undergo intensive, coordinated therapy that supports the restoration of motor function, cognitive abilities, and activities of daily living, thereby facilitating a safer and more prepared transition back home.

### Factors Affecting the Recovery Phase

Recovery after cardiac arrest is influenced by a combination of interacting biological, clinical, psychosocial, and socioeconomic factors. Lin et al. (14) found that a high (neutrophil + monocyte) to lymphocyte ratio (NMLR) is a strong predictor of mortality and poor neurological outcomes. The NMLR value reflects the systemic inflammatory response, which plays a pivotal role in the development of post-cardiac arrest brain injury. The study suggests that assessing simple, readily available biomarkers may assist clinicians in stratifying patients according to risk, thereby informing the required intensity of monitoring and the timely initiation of early rehabilitation interventions.

Alamuti et al. (18) validated the CASPRI, GO-FAR (Good Outcome-Following Attempted Resuscitation), and PIHCA (Prediction of outcome for In-Hospital Cardiac Arrest) scores as accurate clinical prediction tools in IHCA patients. These three scores enable the identification of patients with a good prognosis so that they can be immediately referred to an intensive rehabilitation programme. Conversely, patients with poorer prognostic scores may be better managed with a focus on supportive care and realistic functional recovery goals, tailored to their expected outcomes (18). Meanwhile, Gregers et al. demonstrated that younger age and shorter lengths of hospital stay are strongly related to greater chances of recovery and RTW; these findings emphasised the need for early, as well as prompt detention, diagnosis and treatment stabilisation and efficient post-acute management in improving long-term reintegration outcomes. However, chronic fatigue has been shown to be a major barrier to RTW, even when the patient’s physical condition improves. These findings emphasise that recovery success is not only measured by survival but also by the patient’s ability to resume their role in social and economic life (19).

Wang et al. (16) further identified socioeconomic status (SES) as a significant determinant of outcomes after cardiac arrest. Their findings indicate that patients with lower SES experienced lower rates of ROSC, survival, and neurological recovery compared to those from higher SES backgrounds. These results highlight the critical influence of social determinants such as access to healthcare services, family support systems, and economic stability in shaping long-term recovery outcomes. Meanwhile, Zarifkar et al. (20) highlight neurobiological factors in the form of changes in brain connectivity, particularly in the frontoparietal area, which correlate with cognitive deficits and poor neurological outcomes. However, higher education appears to provide protection against cognitive decline.

Arestedt et al. (17) introduced another important dimension by examining initial clinical characteristics, particularly presenting heart rhythm. They found that patients with non-shockable rhythms (PEA/asystole) at onset were more likely to report lower quality of life and greater psychological distress compared to those with shockable rhythms, indicating that early clinical presentation may have lasting implications for long-term well-being. However, after accounting for other factors such as age and neurological outcome, the influence of initial rhythm weakened. This confirms that initial clinical factors can provide risk indicators, but should not be the sole basis for determining prognosis and recovery pathways.

In line with these findings, Elmer et al. (22) reported that some patients initially assessed as having a poor neurological prognosis ultimately achieved better than expected recovery outcomes. This highlights the considerable variability in post-cardiac arrest trajectories and suggests that existing clinical prediction models may often underestimate patients’ true recovery potential. This finding has significant clinical implications: decisions to discontinue treatment or limit interventions should not be based solely on initial prognostic scores or acute neurological conditions, but should also consider long-term evaluation and the potential for further recovery.

## Conclusion

The recovery phase following cardiac arrest is characterised by a range of multidimensional challenges, including physical, cognitive, psychological, and social difficulties. Many survivors continue to experience persistent fatigue, cognitive impairments, and long-term emotional burdens, such as anxiety, depression, and a diminished sense of self identity. Patients’ families also often face stress and guilt without adequate support. Other studies show that patients with non-shockable rhythms and those with neurocognitive impairments have a lower quality of life. This condition shows that the post-cardiac arrest service system still has a large gap in addressing rehabilitation and psychosocial support needs. Therefore, based on the findings from this mapping and the array of challenges identified, healthcare providers should broaden post-discharge follow-up for cardiac arrest survivors. Care should extend beyond traditional cardiovascular monitoring to encompass a multidimensional recovery approach that addresses physical, cognitive, psychological, and social needs. This approach should include a comprehensive assessment of patients’ cognitive functioning, psychological well-being, and overall quality of life, as well as the emotional and social needs of their families. It is recommended for hospitals to form an integrated follow-up system to which clinical psychologists, occupational therapists and social workers can contribute to ensure that post-recovery needs are identified early on. In addition, it is important to educate patients and families about the recovery journey so that they have realistic expectations and adequate emotional support.

The mapping results also indicate that multiple studies advocate for tackling these challenges in multisectoral, multistakeholder interventions at all levels post-cardiac arrest care. Programmes such as ROCK and SCARF have demonstrated effectiveness in enhancing quality of life, reducing fatigue, and supporting RTW outcomes by integrating physical exercise, cognitive rehabilitation, and psychological support. Peer support also plays an important role in strengthening patients’ self-confidence and emotional adaptation. In addition, structured multidimensional follow-up, routine screening for PICS, and rehabilitation that is sensitive to the patient’s clinical condition have been shown to improve holistic recovery. Therefore, with regard to interventions in the recovery phase, the findings highlight the need to implement comprehensive, evidence-based rehabilitation programmes such as ROCK and SCARF. These programmes have been shown to enhance quality of life, reduce fatigue, and accelerate the restoration of patient independence. Every health facility is encouraged to develop a post-discharge pathway that includes PICS screening, cognitive rehabilitation, psychological support, and peer support groups. The implementation of these interventions can be tailored to the individual conditions of patients through a multidisciplinary approach and cross-professional coordination. The integration of neuroimaging examinations such as fMRI can also be considered to monitor brain function recovery and adjust interventions more appropriately.

The recovery process is further shaped by a range of biological, clinical, and social factors. Prognostic scores including the NMLR and inflammatory biomarkers such as CASPRI, GOFAR, and PIHCA, can help predict neurological outcomes and guide the intensity and focus of rehabilitation interventions. Factors such as age, length of hospital stay, and chronic fatigue also determine the success of RTW. Additionally, low socioeconomic status has been shown to hinder recovery opportunities and survival rates, while changes in brain connectivity are a key determinant of cognitive outcomes in patients. Recent research also indicates that some patients achieve recovery outcomes beyond what initial clinical predictions suggest, highlighting the importance of adopting a more holistic and measured approach to prognosis evaluation. Consequently, policies should promote the use of biomarkers, such as NMLR, alongside clinical prognostic scores, as tools for guiding rehabilitation planning and risk stratification, ensuring that recovery programmes are tailored to each patient’s individual needs. Socioeconomic factors should be carefully considered in service planning, with particular attention to enhancing access to rehabilitation and long-term follow-up for patients with lower income. Strengthening support for these populations can help reduce disparities in recovery outcomes and ensure more equitable post-cardiac arrest care. Community-based approaches and social support need to be optimised to reduce outcome disparities between social groups. In addition, prognosis evaluation should be conducted continuously and not only based on acute conditions, given that some patients are able to recover beyond initial predictions.

## Figures and Tables

**Table 1:** PCC framework

Component	Explanation
P (Population/participant)	Patients who experience cardiac arrest, either IHCA or OHCA; both adults and children.
C (Concept)	Implementation of the recovery phase in the Chain of Survival for cardiac arrest patients.
C (Context)	Health care facilities in hospitals such as inpatient rooms, intensive care unit, paediatric intensive care unit and rehabilitation after ROSC (Return of Spontaneous Circulation) patients, as well as the community and society that play a role after survivors return home from the hospital.

**Table 2 t2-03mjms3301_ra:** JBI critical appraisal tool

References	Study design	JBI quality score (Yes/Total)	Overall methodological quality/risk of bias
Dainty et al. (8)	Qualitative phenomenology	(8/10) 80%	Low risk bias
Nanjayya et al. (9)	Retrospective cohort (data linkage)	(9/11) 81.8%	Low risk bias
Mion et al. (10)	Survey-based descriptive study	Not appraised (no suitable JBI tool)	Not appraised
Wagner et al. (11)	Qualitative study (FGDs)	(8/10) 80%	Low risk bias
Joshi et al. (12)	Prospective one-armed feasibility study (pilot study, non-randomised)	(8/9) 88.9%	Low risk bias
Vincent et al. (13)	Retrospective cohort	(9/11) 81.8%	Low risk bias
Lin et al. (14)	Observational retrospective	(6/8) 75%	Low risk bias
Christensen et al. (15)	Pragmatic Randomised Controlled Trial (RCT)	(10/13) 76.9%	Low risk bias
Wang et al. (16)	Nationwide registry-based observational study	(7/8) 87.5%	Low risk bias
Arestedt et al. (17)	Cross-sectional survey	(6/8) 75%	Low risk bias
Alamuti et al. (18)	Retrospective comparative	(8/10) 80%	Low risk bias
Gregers et al. (19)	Nationwide registry-based cohort study	(9/11) 81.8%	Low risk bias
Zarifkar et al. (20)	Retrospective cohort study	(9/11) 81.8%	Low risk bias

**Table 3 t3-03mjms3301_ra:** Summary of the included studies

Reference	Country	Reported key findings/barriers	Reported key facilitators/implications
Christensen et al. (15)	Denmark	Many survivors experience cognitive, emotional, and social difficulties, leading to 50% failing to RTW.	The ROCK programme (multidisciplinary rehabilitation) supports physical, psychological, and social recovery, improving RTW outcomes.
Dainty et al. (8)	Canada	Survivors report identity loss, fear of relapse, and dependence on families; families feel psychological burden.	Recovery should include psychosocial and relational aspects beyond neurological outcomes.
Joshi et al. (12)	Denmark	High prevalence of post-arrest fatigue and psychological distress.	The SCARF programme combining physical, psychological, and educational therapy, improved fatigue, anxiety, and quality of life.
Nanjayya et al. (9)	Australia and New Zealand	Many patients remain functionally dependent despite rehabilitation gains.	Early rehabilitation improves FIM scores; low baseline FIM predicts better functional improvement.
Vincent et al. (13)	Switzerland	Nearly half of survivors develop PICS symptoms (fatigue, depression, anxiety).	Early multidisciplinary followup, including physical, cognitive, and psychosocial rehabilitation recommended.
Wang et al. (16)	South Korea	Low SES is associated with poorer neurological outcomes and survival.	Equal access to emergency and rehabilitation services is crucial for improving recovery.
Lin et al. (14)	China	High NMLR is linked with higher mortality and poor neurological recovery.	NMLR may serve as a prognostic biomarker for rehabilitation planning.
Arestedt et al. (17)	Sweden	Patients with asystole/PEA rhythms are more likely to experience anxiety or depression.	Psychosocial support and post-resuscitation mental health follow-up are essential.
Alamuti et al. (18)	Iran	Unclear neurological prognosis after IHCA.	CASPRI score shows the highest accuracy for predicting outcomes and guiding rehabilitation focus.
Gregers et al. (19)	Denmark	Return to work rates remain moderate; ECPR did not improve RTW outcomes.	Younger age and shorter hospitalisation predict better recovery and reintegration.
Mion et al. (10)	England	Limited psychological follow-up for survivors and families; cardiology-only care.	Multidisciplinary follow-up including mental health and social support is preferred by survivors.
Wagner et al. (11)	Denmark	Lack of coordinated transition from hospital to community.	Structured rehabilitation and peer support foster coping and adaptation.
Zarifkar et al. (20)	Denmark	Cognitive impairment is common after cardiac arrest despite physical recovery.	Neurocognitive screening and rehabilitation are vital to long-term recovery.

ROCK = return to work after cardiac arrest; SCARF = survivors of cardiac arrest focused on fatigue; FIM = functional independence measure; PICS = post-intensive care syndrome; SES = socioeconomic status; NMLR = neutrophil to monocyte ratio; PEA = pulseless electrical activity; IHCA = in-hospital cardiac arrest; CASPRI = cardiac arrest survival post-resuscitation in-hospital; ECPR = extracorporeal cardiopulmonary resuscitation; RTW = return to work
